# Impact of using creative arts programming to support HIV treatment in adolescents and young adults in Eswatini

**DOI:** 10.1186/s12981-021-00423-2

**Published:** 2021-12-20

**Authors:** Tara E. Ness, Vedika Agrawal, Danielle Guffey, Amanda Small, Tandzile Simelane, Sandile Dlamini, Jaime Petrus, Bhekumusa Lukhele

**Affiliations:** 1 Baylor College of Medicine, Houston, TX USA; 2 Texas Children’s Hospital, Pediatric House Staff Office, 6621 Fannin St, Houston, TX 77030 USA; 3 Baylor Center of Excellence, Mbabane, Eswatini

**Keywords:** Adolescents, HIV, Social support, Performing arts, Program evaluation

## Abstract

**Background:**

In 2018, approximately 1.6 million adolescents (aged 10–19) were living with HIV worldwide, with the highest HIV prevalence found in Eswatini. Adolescents and young adults living with HIV are a vulnerable population due to unique psychosocial challenges that come with having a stigmatizing disease. This group struggles more than other age-groups with medication adherence and requires novel approaches to supporting treatment, including peer-group encouragement, and self-expression.

**Methods:**

We piloted a theater camp for a group of adolescents and young adults enrolled at our HIV clinic in Mbabane, Eswatini, to determine the impact of having an outlet for creative expression and peer support on treatment and feelings of stigma. Pre- and post-camp surveys were administered to the participants to assess perceived stigma and impact of the camp. The results were analyzed using a Wilcoxon-signed rank test.

**Results:**

Twenty individuals (ages 12–23) living with HIV participated in the camp concurrently with standard treatment. 25% showed a substantial decrease in viral load within six months of completing the camp (> 0.1 log_10_ change) while only 10% showed a substantial increase. Those who completed the survey felt the camp helped them with confidence, teamwork, and friendships. A comparison of pre- and post- surveys showed an overall decrease in personalized stigma. Quotes from participants reinforced these results.

**Conclusions:**

Adolescents and young adults living with HIV are an important population for further program development. Our study showed creative arts programming has beneficial psychosocial effects, aids in community building, and potentially enhances the effectiveness of medical treatment. Further programs and studies should continue to investigate creative arts as an avenue for self-expression and community building among vulnerable populations.

**Supplementary Information:**

The online version contains supplementary material available at 10.1186/s12981-021-00423-2.

## Background

In 2018, approximately 1.6 million adolescents (aged 10–19) were living with HIV/AIDS (human immunodeficiency virus)/ (acquired immunodeficiency syndrome) worldwide, with about 89% of them being located in sub-Saharan Africa [1]. High rates of new infections (approximately 190,000 worldwide per year) and the failure of prevention of mother to child transmission (PMTCT) due to limited access to antiretroviral (ARV) medication contribute to these numbers [1]. In the kingdom of Eswatini, the rate of mother to child transmission ranged from 25 to 40%, encompassing pregnancy, birth, and breastfeeding, prior to PMTCT becoming widely available in 2003 [2, 3]. Despite the significant progress that has been made in the availability of ARVs, adolescents and young adults with HIV continue to be an important population with unique psychosocial challenges due to their age. Addressing these issues requires creative approaches to treatment and prevention that both empower adolescents and young adults and provide them with skill sets to actively participate in society.

Despite having one of the highest rates of antiretroviral coverage in the region (86%) for people living with HIV (PLHIV), Eswatini also has the highest prevalence of HIV in the world at 27.3% [4]. While in 2010 there were 1,600 new infections in children (age 0–14 years), in 2017 this had reduced to less than 1,000 new infections per year [4]. Working against the substantial progress that has been made is significant stigma and discrimination surrounding HIV, exemplified by 37% of both men and women displaying discriminatory attitudes towards PLHIV [5] and the 2011 Stigma and Discrimination index finding that self-stigma among PLHIV was even higher than externally expressed stigma [6]. Stigma can have a significant impact on people presenting for testing and treatment, as well as medication adherence and appropriate clinic follow up [5].

Adolescents and young adults living with HIV in Eswatini make up an important demographic for treatment support. Despite the fact the WHO highlighted the urgent need for tailored HIV services for adolescents in 2013 [7], there is minimal previous research exploring distinctive approaches to treatment support specifically in this population [8].

Here, as well as other places, clinics have tried to come up with novel approaches to support empowerment and community building amongst teens to improve retention in care and adherence to therapy. Some, including ours, have adopted the use of “Teen Clubs” where adolescents regularly meet outside of clinic hours and participate in educational and bonding activities. Certain studies have shown an increased retention in care, particularly among older adolescents [9, 10], and attendance has been linked to viral suppression [11], however the evidence is inconclusive. In Eswatini, specifically, Teen Club was evaluated and reported to improve adherence, promote disclosure, and empower adolescents in overcoming stigma [12]. Other approaches, such as recreational therapy camp, have been shown to have a short-term increase in adherence in the six months following camp participation [13].

Our group sought to trial a unique approach to treatment support through the use of creative arts programming combined with some of the community-support elements of aforementioned programs. Through the use of music and theater education, we aimed to encourage self-expression, creativity, and empowerment amongst adolescents and young adults enrolled in our clinic. This could also promote a sense of community within the group as they all work towards a common goal and to develop skills in public speaking, story development, cooperation, problem solving, and leadership.

While previously implemented programs have utilized counseling, education, and community-support to increase retention in care and adherence to medication, our intervention aims to explore the utility of creative arts programming in addressing the distinct psychosocial challenges adolescents and young adults face living with a stigmatizing condition at their age. We believe that addressing these factors contributing to poor retention and adherence rates among this age group will also improve retention and adherence themselves. We hope by publishing our results other institutions will consider creative arts programming to build camaraderie and friendship among adolescents and young adults with chronic, often stigmatizing conditions, and build upon our intervention with other ideas for tackling this specific population.

## Methods

### Study population

Adolescents and young-adults between the ages of 13–25 years with HIV that were actively enrolled at the Baylor College of Medicine- Bristol-Myers Squibb Children's Clinical Centre of Excellence in Mbabane, Eswatini were invited to participate, with emphasis on the enrollment of those struggling with adherence, teen mothers, or those on second-line antiretroviral treatment. This allowed us to target participants that were aware of their HIV status (disclosure generally occurring between 10 and 13 years of age) and encompass both adolescents and young adults, who are identified as facing unique psychosocial challenges in regard to their HIV diagnosis. Exclusion criteria were that the participant had not yet had their HIV status disclosed by their caregiver(s), were unable to provide written permission for participation from their caregiver(s) or whose participation was required in the traditional Swazi ceremony of Umhlanga, which is a culturally significant annual event in Eswatini.

### Compliance with ethical standards

This project was approved by the clinic executive director in Mbabane, Eswatini as well as the Baylor International Pediatric AIDS Initiative (BIPAI) headquarters in Houston, Texas. The project proposal was submitted and approved with determination that the project fell within the ‘*MEDICAL AUDIT OF PATIENTS REGISTERED AT THE BAYLOR COLLEGE OF MEDICINE CHILDREN’S FOUNDATION—SWAZILAND CLINICAL CENTRES OF EXCELLENCE’* protocol annually approved by the Baylor Institutional Review Board. Prior to camp, all theater camp participants provided verbal consent to participation and their caregivers provided written consent and acknowledgement of camp activities the adolescents and young adults would be participating in. The study was performed in accordance with the ethical standards as laid down in the 1964 Declaration of Helsinki and its later amendments or comparable ethical standards.

### Theater camp curriculum

In collaboration with a non-profit organization of professional teachers, actors, and musicians (omitted to protect participants anonymity and HIV status), a two-week theater camp was conducted in Mbabane, Eswatini. To allow participants of all income levels to participate, transport money to and from camp, breakfast, and lunch were provided. The camp ran from 8 am to 3 pm Monday through Friday at a school facility while regular classes were not in session. Camp participants gathered each morning and worked with the teachers on various activities as part of the theater camp curriculum, including group bonding with games, prompt-based improvisation, short skits, lyric writing, and making speeches in front of the group. The main curriculum involved supporting camp participants in developing an original plot and creating music and lyrics to go along with the story as a way to foster camaraderie, friendship, and community amongst the group as well as empower self-expression. For example, small groups of participants would be given a prompt and encouraged to come up with a scene and characters to then perform for the group. They practiced how to read and react to each other’s actions and worked on how to perform for an audience. Over the camp session, various scenes from the improvised skits were selected and woven together to create a story of two brothers navigating the world after the loss of their parents at a young age. Campers worked together to come up with song lyrics to reflect themes of the play and several original compositions were put together and rehearsed. At the end of two weeks, the campers performed their play on a local theater stage for their family, friends, and clinic staff. The theme of the play the participants developed did not directly address HIV.

### Questionnaires (including pilot survey)

We put together a questionnaire using a brief version of the HIV Stigma scale [14, 15] in addition to four questions pertaining to future outlook, and four questions pertaining to medication adherence motivation. The questionnaire can be found under Additional file 1: Fig. S1. All questions were based on a 5-point Likert scale. The survey was provided in both English and siSwati language and was voluntary.

The HIV Stigma Scale is a questionnaire that consists of questions that address personalized stigma, disclosure concerns, concerns about public attitudes towards HIV, and negative self-image [14, 15] with the brief version of the scale consisting of twelve questions that cover these same domains but with less questions to avoid survey fatigue [16]. The original survey has a high internal consistency (α = 0.96) and good test–retest probability (*r* = 0.92) with the shortened version of the scale having a high Cronbach alpha value (> 0.7) for all subscales.

We performed a pilot survey with our questionnaire to validate it in our population at a Teen Club a few weeks prior to camp starting, with individuals who were consistent with our inclusion/exclusion criteria and therefore representative of our camp participants. None of the individuals who completed pilot surveys went on to participate in the camp.

On the first and last day of camp, the questionnaire was given to all the camp participants to complete anonymously. In addition, on the last day a qualitative survey was provided to the campers providing an opportunity for additional feedback of their experience.

### Biological data

For each camp participant, data regarding age, gender, current antiretroviral medication (including whether it was first, second, or third line), latest CD4 count (within 12 months), and viral load preceding and post camp within 6 months was extracted from the electronic medical record. Information was de-identified, and each participant was given a unique code for identification.

### Data analysis

All data were entered into Microsoft Excel for organization and analyzed using Microsoft Excel or STATA. Patient characteristics were summarized using mean with standard deviation, median with 25th and 75th percentiles, and frequency with percentage. Cronbach's alpha was used to measure the internal consistency of the HIV Stigma Score for all patients and by survey language. Pre- and post-theater camp scores were compared using Wilcoxon signed rank test for all patients by survey language. The questions were additionally regarded as binary “agree” and “not agree” and compared pre- and post-theater camp using McNemar's test for all patients by language the survey was taken in. Viral load data was summarized with a > 0.1 log_10_ change considered significant.

## Results

### Participant demographics

Twenty-three individuals with HIV completed pre-camp surveys. Twenty of those went on to participate in the theater camp. Of those twenty, there were six females and fourteen males, and their ages ranged from 12 to 23. The youngest female was 16 with the majority of females (five) between the ages of 20 and 22. Twelve individuals were on second line ARVs and eight individuals were currently enrolled in “Challenge Clinic”- a specialized clinic program for those with significant social issues and/or detectable viral loads on second-line ARV regimens. CD4 counts ranged from 15 to 1580, with a mean of 665 and were all taken within the twelve months preceding camp. Six individuals had detectable viral loads, ranging from log 2.37 (234 copies) to log 5.46 (285,361 copies). Table 1 summarizes the demographic distribution of the 20 camp participants. The three individuals who completed the pre-camp survey (two females, one male; two 19-year-olds and one 23-year-old) but did not ultimately continue with the camp did not state their reason for lack of participation.

**Table 1:** Demographics of camp participants

Demographic value		Frequency			
		Number	Percentage	Median	Q1–Q3
Gender					
Male	14		70%	–	–
Female	6		30%	–	–
Age					
12–17	10		50%	17.5	14–20
18–23	10		50%		
ARV regimen					
First-line	8		40%	–	–
Second-line	12		60%	–	–
Challenge clinic					
Enrolled	8		40%	–	–
Not Enrolled	12		60%	–	–
Pre-camp viral load					
Detectable	6		30%	Log 1.3 (20 copies)	Log 1.3–2.6 (20–525.5 copies)
Not Detectable	14		70%		
CD4 Count					
< 400	4		20%	623	535.25–802
> 400	16		80%		

### Questionnaires

Twenty-two individuals completed the pilot survey to validate the survey in our specific population, 10 in English and 12 in siSwati. This was a separate group of individuals from those that participated in our theater camp but would have met inclusion and exclusion criteria. Of the 22 pilot survey participants, 7 were male, 13 were female, and 2 did not state their gender. For the Brief HIV Stigma Scale (questions 1–12), there was acceptable internal consistency (α > 0.7) for both those who took the survey in English (α = 0.78) and siSwati (α = 0.71). Questions pertaining to future and medication adherence (separate from the Brief HIV Stigma Scale) were noted to have unacceptable internal consistency (α < 0.5) so were removed from further analysis.

Twenty individuals participated in the theater camp. Of the twenty camp participants, nineteen participants (six females and thirteen males) completed both pre- and post- camp surveys (95% response rate). The one male who did not complete the post-camp survey was unavailable at the time it was administered. Analysis of the survey responses pre- and post-camp showed less agreement with the statement “I am very careful who I tell that I have HIV” regarding disclosure concerns after the camp (p = 0.046) as well as decreased personalized stigma (questions 1–3, p = 0.025). No other significant differences in questions or scales were found when controlled for gender, age, or survey language. When questions were analyzed with grouping of similar Likert scale answers (binary “agree” or “disagree”) there were no further significant differences discovered.

Eighteen individuals who completed the post-camp survey also completed the non-Likert survey. Of these, 72% stated camp helped them with confidence (13/18) and teamwork (13/18), and 61% (11/18) stated it helped them with friendships. 100% of the respondents stated they would participate in the theater camp again if given the opportunity. When asked what they liked about theater camp, one participant wrote they liked “The fact that there was no discrimination between us,” and another stated “It made me not feel sorry for being an HIV positive person.” Table 2 lists additional participant responses arranged by theme with minor grammar editing.

**Table 2:** Quotes from camp participants in response to the question “What did you like about theater camp?”

Theme	Participant Quote
Confidence	“Theater camp helped me a lot. It taught us to be brave and confident in what we do.”
	“Theater camp helps me know that I have a talent and I can do a lot of things in life to achieve my future goals although I am HIV positive.”
	“Theater camp helped me with being brave.”
Skills	“What I liked about theater camp is they teach you public speaking.”
	“Theater camp has helped me in many ways. Sharing ideas and opinions to tell a story.”
	“Self-expression, realizing my emotions, storytelling techniques.”
Community/teamwork	“Progress is faster when two or more people are synced as a team.”
	“The thing I loved most is that I made friends and felt comfortable around people who I have the same (HIV) status.”
	“I liked the fact that I met young adults who are like me and having them in my corner.”

### Pre- and post-viral load monitoring

Viral loads were available six months pre- and post-camp for nineteen of the twenty theater camp participants (one participant has not had a follow up viral load after camp). Five individuals (25%) had a significant decrease in viral load from before camp while only two individuals (10%) had a significant increase. The average viral load pre-camp was 17,767 (log 4.24) and the average viral load post-camp was 795 (log 2.9). The two individuals who had a significant increase in post-camp viral load went from undetectable pre-camp to log 2.66 and log 3.29.

## Discussion

This study suggests potential beneficial effects for adolescents and young adults living with HIV that participate in creative arts programming such as theater. Our most significant results included a decrease in personalized stigma from pre- and post-camp surveys as well as a decrease in wanting to withhold disclosure of HIV status to others. Participant responses asking more specifically about impact of the camp in terms of teamwork, friendship, and community showed a substantial positive impact and participants readily provided quotes in our qualitative survey about the benefits of camp. The positive impact on viral load, seen both by the overall decrease in average viral load and in the number of participants with decreased vs increased viral load was especially noteworthy. Although our intervention was brief (two weeks), it has laid the foundation for further projects that are more longitudinal in nature and with the capacity to involve more participants. As the HIV community works towards the 95–95-95 strategy set forth by UNAIDS (Joint United Nations Programme on HIV and AIDS) to ensure 95% of those with HIV are diagnosed, 95% of those diagnosed are on ARVs, and 95% are suppressed [17], creative approaches to helping adolescents and young adults meet these goals will be required. Our intervention, specifically, helps with decreasing personal stigma that may prevent individuals from getting tested and supporting viral load suppression.

The decrease in reported personalized stigma was significant and, based on participant quotes, may have been due to the camp creating a community of individuals with a similar HIV status working towards a common goal. By creating a safe space of self-expression and providing tools for self-expression, the participants became more comfortable and formed bonds with one another in a short span of time. The improvement in viral load, often used to monitor medication adherence and engagement in therapy, was extremely encouraging considering many of the camp participants struggled with adherence prior to camp. We believe this is in part due to the self-empowerment provided through self-expression and self-confidence from creating a successful piece of art and sharing it with their community. The quotes provided by the campers express they gained important skills from participating, made friendships, and learned how to work as a team towards a common goal. Both quantitatively (via viral load) and qualitatively (via survey responses) there was a significant impact of the camp on participants. It is unclear whether these same results could be seen with other forms of creative arts.

According to the WHO, “Adolescents living with HIV have worse access to antiretroviral treatment, adherence to treatment, retention in care and viral suppression. A key factor contributing to these is limited provision of adolescent-friendly services including psychosocial interventions and support [18].” Research examining creative art therapies has primarily been in the realm of mental illness treatment and, although results on its effectiveness vary and are sometimes contradictory, it has shown promise in reducing disease-specific symptoms and improving mental health and overall well-being [19]. Drama interventions in particular, though among the ones less studied, have been shown to improve self-esteem, feelings of inferiority, social functioning, and emotional expression in schizophrenic patients [19–21]. Although we were unable to find any previous studies that attempted a similar intervention utilizing creative arts (e.g. theater, sculpture, painting) as a means of treatment support in HIV/AIDS, there were a few other interventions specifically aimed at adolescents and young adults in the form of “Teen Clubs.” These meetings occur outside of regular clinic hours with some studies showing greater retention in care [9, 10]. In Eswatini, Teen Club was evaluated and reported to improve adherence, promote disclosure, and empower adolescents in overcoming stigma [12]. Our intervention required partnership between a medical clinic and those with expertise in performance arts, which are fields that do not usually have a lot of overlap. In addition, this kind of intervention is difficult to objectively assess for impact. Regardless of these obstacles, it is worthwhile to identify and attempt novel interventions to support treatment in at-risk or struggling populations to augment medical management.

There are a number of limitations to our study worth mentioning. First and foremost, our camp was completely voluntary, so participation in the camp may have provided bias in responses to survey questions after camp conclusion that were affirmative because of this unintended selection. That being said, there was still a significant change in attitude of personal stigma in this group with our structured survey pre- and post-camp, showing that even with a group with possible selection bias there is improvement seen. Another limitation of our camp is that we do not have a proper control group to compare viral load trends during the same period to assess if the positive impact on viral suppression was seen in the general clinic population as well. Because of the nature of our intervention, we were also limited by a small sample size, which limited our power and sensitivity. Another thing to note is that our theater program did not specifically address HIV. It is possible that the intervention did not show stronger effects on perceived stigma as the camp itself did not directly focus on or discuss HIV unless brought up by the participants themselves. While it was clear to all participants that they all shared this condition, this group chose not to bring HIV up in any of the camp activities, although they may have spoken about it amongst themselves. It is possible specially designed programs that address HIV directly could have an even stronger impact in this population than we have seen so far.

## Conclusions

Adolescents and young adults living with HIV are an important population for further program development. Overall, our study shows creative arts programming, even when not directly addressing HIV, has beneficial psychosocial effects, aids in community building, and potentially enhances the effectiveness of medical treatment. This work is an important step in specifically addressing adolescents and young adults with HIV and coming up with creative approaches to their treatment support, as advocated by UNAIDS and others. Further programs and studies should continue to investigate creative arts as an avenue for self-expression and community building among vulnerable populations.

## Supplementary Information

**Additional file 1: Fig. S1**. English version of survey questions provided to camp participants. Each question designated “Please circle an answer to each of the below questions based on your experiences” with a 5-point Likert scale.

## Data Availability

The dataset used and/or analyzed during the current study are available from the corresponding author on reasonable request.

## Abbreviations

AIDS: Acquired immunodeficiency syndrome
BIPAI: Baylor International Pediatric AIDS Initiative
ARV: Antiretrovirals
HIV: Human Immunodeficiency Virus
PLHIV: People living with HIV
PMTCT: Prevention of mother to child transmission
UNAIDS: Joint United Nations Programme on HIV and AIDS
WHO: World Health Organization
