# Supplementary material for: Impact of using creative arts programming to support HIV treatment in adolescents and young adults in Eswatini
Source: AIDS Res Ther. 2021 Dec 20;18:100. doi: 10.1186/s12981-021-00423-2 (PMC8690402; doi:10.1186/s12981-021-00423-2)
Supplement: Supplementary file 1 — Additional file 1: Fig. S1. English version of survey questions provided to camp participants. Each question designated “Please circle an answer to each of the below questions based on your experiences” with a 5-point Likert scale. [file 12981_2021_423_MOESM1_ESM.docx]

**Supplementary Figure I**

Brief HIV Stigma Scale

**Personalized stigma**

Some people avoid touching me once they know I have HIV

People I care about stopped calling after learning I have HIV

I have lost friends by telling them I have HIV

**Disclosure concerns**

Telling someone I have HIV is risky

I work hard to keep my HIV a secret

I am very careful who I tell that I have HIV

**Concerns about public attitudes**

People with HIV are treated like outcasts

Most people believe a person who has HIV is dirty

Most people are uncomfortable around someone with HIV

**Negative self-image**

I feel guilty because I have HIV

People’s attitudes about HIV make me feel worse about myself

I feel I’m not as good a person as others because I have HIV

Future Outlook Questions

I feel ready to tell someone that I have HIV

I am excited for my future

I feel that I have a community of people to support me in managing my HIV in the future

I feel that HIV will not hold me back from achieving my dreams

Medication Adherence Motivation Questions

I feel that taking my medications is important for my health

I am motivated to take my medicines every day

If I am considering not taking my medicines, I would reach out to a friend

If I had a friend that stopped taking their medicine, I would encourage them to restart

English version of survey questions provided to camp participants. Each question designated “Please circle an answer to each of the below questions based on your experiences” with a 5-point Likert scale.
